# PCR targeting *Plasmodium* mitochondrial genome of DNA extracted from dried blood on filter paper compared to whole blood

**DOI:** 10.1186/1475-2875-13-137

**Published:** 2014-04-07

**Authors:** Gro EA Strøm, Sabrina Moyo, Maulidi Fataki, Nina Langeland, Bjørn Blomberg

**Affiliations:** 1 Department of Clinical Science, University of Bergen, Bergen, Norway; 2 National Centre for Tropical Infectious Diseases, Department of Medicine, Haukeland University Hospital, Bergen, Norway; 3 Muhimbili University of Health and Allied Sciences, Dar es Salaam, Tanzania; 4 Muhimbili National Hospital, Dar es Salaam, Tanzania; 5 Department of Medicine, Haukeland University Hospital, Bergen, Norway

**Keywords:** Malaria, PCR, Filter paper, Dried blood spots/DBS, Chelex, InstaGene Matrix

## Abstract

**Background:**

Monitoring mortality and morbidity attributable to malaria is paramount to achieve elimination of malaria. Diagnosis of malaria is challenging and PCR is a reliable method for identifying malaria with high sensitivity. However, blood specimen collection and transport can be challenging and obtaining dried blood spots (DBS) on filter paper by finger-prick may have advantages over collecting whole blood by venepuncture.

**Methods:**

DBS and whole blood were collected from febrile children admitted at the general paediatric wards at a referral hospital in Dar es Salaam, Tanzania. DNA extracted from whole blood and from DBS was tested with a genus-specific PCR targeting the mitochondrial *Plasmodium* genome. Positive samples by PCR of DNA from whole blood were tested with species-specific PCR targeting the 18S rRNA locus, or sequencing if species-specific PCR was negative. Rapid diagnostic test (RDT) and thin blood smear microscopy was carried out on all patients where remnant whole blood and a blood slide, respectively, were available.

**Results:**

Positivity of PCR was 24.5 (78/319) and 11.2% (52/442) by whole blood and DBS, respectively. All samples positive on DBS were also positive on *Plasmodium falciparum* species-specific PCR. All RDT positive cases were also positive by DBS PCR. All but three cases with positive blood slides were also positive by DBS.

**Conclusions:**

In this study, PCR for malaria mitochondrial DNA extracted from whole blood was more sensitive than from DBS. However, DBS are a practical alternative to whole blood and detected approximately the same number of cases as RDTs and, therefore, remain relevant for research purposes.

## Background

Malaria killed approximately 627,000 people, mostly children in sub-Saharan Africa, in 2012 and remains an endemic disease in 97 countries. Globally it is a major health concern, especially in low-resource settings [1].

The diagnosis of malaria is challenging and the conventional, gold standard, diagnostic method using blood smear microscopy lacks sensitivity and specificity in many malaria-endemic settings [2-4]. Polymerase chain reaction (PCR) is a molecular technique that is often used for research purposes [5,6]. Conventionally, PCR has been performed on DNA extracted from whole blood. However, PCR amplification of *Plasmodium* DNA extracted from dried blood spots (DBS) on filter paper, rather than from whole blood, is a method that can be very useful in low-resource settings and is being used increasingly [7]. The use of DBS allows material to be collected in rural areas and stored at room temperature without the need of a cold chain. DBS require less blood than when whole blood is used for PCR and can be collected by finger-prick, which is minimally invasive compared to conventional methods of whole blood collection by venepuncture. With falling numbers of malaria cases [8] and emerging artemisinin resistance [9,10], precise malaria diagnostics and monitoring of malaria transmission is of increasing importance. Also, with a goal of malaria elimination becoming more relevant [1], monitoring malaria epidemiology, including submicroscopic quantities of malaria detected only by PCR [11,12], is necessary. It has been shown in some studies that PCR on DNA extracted from filter paper is less sensitive than PCR done on DNA extracted from whole blood [13], but the discrepancies found are not very remarkable. Ataei *et al.* found 42.7% positive for *Plasmodium* species by PCR on filter paper and 53.3% positive on whole blood [7], while a study by Proux *et al.* showed a smaller reduction in malaria detection by DBS as compared to whole blood [14]. Another study showed a ten- to 100-fold decrease in sensitivity when using DBS as compared to whole blood [15].

The aim of this study was to compare detection of malaria by *Plasmodium* PCR of DNA extracted from DBS and from whole blood. Additionally, positivity of PCR from the two extraction methods and results of species-specific PCR, RDT and microscopy were compared.

## Methods

### Data collection

A total of 469 febrile children admitted to the general paediatric wards at Muhimbili National Hospital in Dar es Salaam, Tanzania in 2009 were included in the study after informed, written consent was obtained from the child’s parent or guardian by signature or thumbprint. DBS were obtained from 442 of the study participants and they were prepared by dropping two drops of venous blood, collected with a syringe at the same time as other blood tests were taken, on a segment of a Whatman^®^ Schleicher & Schuell filter paper, grade 589/2 (Whatman GmbH, Dassel, Germany). The DBS was stored in a sealed, airtight plastic pocket after air-drying completely. The DBS were transported and stored protected from sunlight and after initial storage at approximately 25°C for three to nine months they were stored for about three-and-a-half years at -20°C until DNA extraction was performed [16,17]. A vial of venous blood with EDTA was only obtained for the study from 319 of the 469 included children (of which 303 also had DBS available) and the remaining content in the vial after routine haematological tests were performed was stored for two to two-and-a-half years at -20°C for ensuing DNA extraction. DBS and whole blood were missing from some of the patients due to practical and communicational challenges encountered during the data collection. DBS and EDTA blood vials were transferred to the University of Bergen using the Material Transfer Agreement of Muhimbili University of Health and Allied Sciences (MUHAS) and the EDTA blood samples were kept frozen using dry ice during transport.

### DNA extraction

Harris Uni-Core^TM^ puncher (Qiagen, Hilden, Germany) was used to punch out six pieces (or fewer if fewer than six punches were available) of filter paper with dried blood 3 mm in diameter. The puncher was cleaned as described elsewhere (Strom GE, Fataki M, Langeland N, Blomberg B: Comparison of four methods for extracting DNA from dried blood on filter paper for PCR targeting the mitochondrial *Plasmodium* genome, unpublished) and blank filter paper pieces punched out in the last step of the washing process were subjected to DNA extraction, followed by PCR between random samples to ensure no transfer of parasite DNA between samples using this cleaning method.

DNA was extracted from the DBS using a method with Chelex-100^®^ Molecular Biology Grade Resin (Bio-Rad Laboratories, Hercules, CA, USA) and soaked in 0.5% saponin in phosphate buffered saline (PBS) solution overnight, as previously described as the method with the lowest limit of detection (0.5 parasites/μl) among the currently most used methods for DNA extraction from DBS (Strom GE, Fataki M, Langeland N, Blomberg B: Comparison of four methods for extracting DNA from dried blood on filter paper for PCR targeting the mitochondrial *Plasmodium* genome, unpublished). DNA was also extracted from 200 μl whole blood (venous blood collected in a vial with EDTA anti-coagulant) using QIAamp^®^ DNA Blood Mini Kit (Qiagen, Hilden, Germany) as described by the manufacturer, and eluted in a final volume of 100 μl.

### PCR

A genus-specific PCR targeting *Plasmodium* mitochondrial genome, as described by Haanshuus *et al.*[18], but with a primer concentration of 1 μM, was performed on DNA extracted from whole blood. The PCR method used on the DBS was the same as above but employed the producer-recommended HotStarTaq Plus DNA polymerase (Qiagen, Hilden, Germany) and CoralLoad PCR buffer (Qiagen, Hilden, Germany), and therefore step one at 95°C in the cycling parameters had a duration of five rather than 15 minutes. The final PCR reaction volume was 20 μl including 2 μl template. Amplification was done using GeneAmp PCR System 9700 (Applied Biosystems, Carlsbad, CA, USA). For species identification, species-specific PCR targeting the 18S gene for *P. falciparum*, *Plasmodium vivax*, *Plasmodium malariae* and *Plasmodium ovale* was done on all whole blood samples positive by genus-specific PCR as described by Haanshuus *et al.*[18]. Those negative by species-specific PCR were sequenced. Analysis was done by electrophoresis using 2% SeaKem^TM^ agarose gel (Lonza, Rockland, ME, USA) with 1X GelRed^TM^ (Biotium, Hayward, CA, USA).

Slides for research microscopy were obtained for 403 of the patients and enough blood remained to perform RDT for 271 study participants. Microscopy and RDTs were performed as described elsewhere [2].

### Ethics

A research permit was obtained from the Tanzania Commission for Science and Technology (COSTECH), and ethical clearance was received from the appropriate bodies at MUHAS and MNH and from the Regional Committee for Medical and Health Research Ethics, Western Norway. The study was done in collaboration between MUHAS/MNH and the University of Bergen/Haukeland University Hospital, Norway.

## Results

Of the DBS samples, 52/442 (11.2%) were positive, as compared to 78/319 (24.5%) of the whole blood samples. Of those positive by PCR on whole blood, 46.1% (35/76) were negative on filter paper. All patients positive on PCR of DNA extracted from DBS were also positive on PCR for *P. falciparum* for patient samples where PCR on whole blood was done*.* None was only positive by sequencing. No other *Plasmodium* species were detected other than *P. falciparum*. All those positive by RDT (36) were also positive by PCR of DBS. However, one case was positive on PCR of DBS that was negative by RDT. Of those negative on PCR from DBS and where research blood smears were available, three (0.7%) were positive on research microscopy and one of these was also negative on whole blood PCR. PCR of DNA extracted from DBS detected 25 more positive cases than were detected by research microscopy (22 positive cases).

Sensitivities, specificites, positive predictive value (PPV), and negative predictive value (NPV) of PCR on DNA from DBS, research microscopy and RDT when compared to PCR of DNA from whole blood are found in Table 1.

**Table 1:** Sensitivity, specificity, PPV and NPV of PCR of DBS, study microscopy and RDT with PCR of whole blood as a gold standard

**Diagnostic method**	**Sens (%)**	**Spec (%)**	**PPV (%)**	**NPV (%)**
**DBS PCR**	41/76 (53.9)	227/227 (100.0)	41/41 (100.0)	227/262 (86.6)
**Study micro**	20/76 (26.3)	227/228 (99.6)	20/21 (95.2)	227/283 (80.2)
**RDT**	38/71 (53.5)	199/199 (100.0)	38/38 (100.0)	199/232 (85.8)

*PPV*: positive predictive value; *NPV*: negative predictive value; *PCR*: polymerase chain reaction; *DBS*: dried blood spot; *RDT*: rapid diagnostic test for malaria; *Sens*: sensitivity; *Spec*: specificity; micro: microscopy.

## Discussion

The study aimed to compare *Plasmodium* genus-specific PCR of DNA extracted from whole blood and DNA extracted from filter paper (DBS) taken from febrile children admitted to general paediatric wards at a referral hospital in Tanzania. The number of participants positive by PCR of DNA from whole blood was almost twice as many as those positive by DNA from DBS. This was surprising given the low limit of detection of both the extraction method (Strom GE, Fataki M, Langeland N, Blomberg B: Comparison of four methods for extracting DNA from dried blood on filter paper for PCR targeting the mitochondrial *Plasmodium* genome, unpublished) and of the PCR method used [18]. The Chelex-100^®^-based method for DNA extraction from DBS used in this study previously proved to be the method for DNA extraction from DBS with the lowest limit of detection when compared to several other commonly used extraction methods (Strom GE, Fataki M, Langeland N, Blomberg B: Comparison of four methods for extracting DNA from dried blood on filter paper for PCR targeting the mitochondrial *Plasmodium* genome, unpublished). It is a method that is cost-effective and simple and has also previously been used in many malaria-studies using DBS [16,19-23].

All those positive by PCR of DBS were also positive on *P. falciparum* PCR targeting the 18S rRNA gene. The mitochondrial genome is present in more copies per parasite than the 18S gene [18]. This may indicate that the cases positive on DBS were cases with higher parasitaemia with enough copies of the 18S locus as well as the mitochondrial genome to result in both positive genus- and species-specific PCR. The conventional single-amplification PCR used was chosen as it previously has been performed non-inferiorly to a reference nested-PCR and is less time-consuming and previously has performed well [2,18] (Strom GE, Fataki M, Langeland N, Blomberg B: Comparison of four methods for extracting DNA from dried blood on filter paper for PCR targeting the mitochondrial *Plasmodium* genome, unpublished).

When using DBS rather than whole blood as a source of DNA for PCR the concentration of DNA is much lower. In six 3-mm punches of DBS there is approximately 25 μl blood (a 50 μl DBS gives an average 12 punches per DBS), which, using the DNA extraction method used in this study, ends in a final elution volume of approximately 80 μl template. Using the QIAamp^®^ DNA Blood Mini Kit, 200 μl whole blood resulted in a final elution volume of 100 μl. This results in more than five times greater DNA concentration using the whole blood method compared to the DBS method. This is likely an important contributing factor to the lower sensitivity of the DBS method as compared to the whole blood method.

Storage of DBS has previously been shown to influence the sensitivity of PCR performed on extracted DNA. One study reported sensitivity to decrease after six years [24] and the samples used in the current study had been stored for four to four-and-a-half years before analysis. However, another study actually showed an increased sensitivity after storage of DBS for more than four years [25] though these results have not been reproduced and the study was done without using proper controls. Also this increased sensitivity was no longer present after purification steps were repeated of the more recently collected samples. Not many studies have been done comparing PCR of whole blood and filter paper, but many studies have used only DBS as a source of DNA for PCR of participants.

The use of DBS is practical for studies of anti-malarial resistance genes [26], anti-malarial drug concentrations in blood [27] and for reliable species identification as well as monitoring malaria epidemiology, including parasitic genotyping to consider whether return of parasitaemia after a short time is due to re-infection or recrudescence [28]. It detects more cases than microscopy, although the sensitivity found in this study, with PCR of DNA extracted from whole blood as the gold standard, was not much higher than for RDTs (53.9% (Table 1), and 52.9% (Additional file 2 of reference [2]), respectively). A study done in Senegal in an urban setting, showed a higher discrepancy between DBS and microscopy results with 15.2% of asymptomatic study participants positive by PCR of DBS but negative on thick blood smear microscopy [29]. The sensitivity of the RDT in this study was better than previously shown in a similar comparison of PCR of DBS and RDT by Fancony *et al.*[30]. DBS have the advantage that they provide DNA, which can be analysed for the above-mentioned additional purposes. The current study is representative of how field conditions may truly be, with high temperatures and challenging DBS preparation and storage conditions. This may also have contributed to the lower than expected sensitivity of PCR on DBS as high temperatures initially during storage may have reduced DNA quality [15]. The applicability of the results of the study is broad and realistic in a resource-poor, malaria-endemic setting. The fact that different methods of DNA extraction, PCR and RDTs have been used in the various studies reduces the value of a detailed comparison.

## Conclusion

Using the methodology followed in this work, the sensitivity of PCR for malaria on DNA extracted from DBS is approximately half of the sensitivity of PCR on DNA extracted from whole blood. Nonetheless the advantages of DBS when it comes to storage and transport may to some degree compensate for this when applying the use of DBS for research in rural settings where collection of whole blood is not possible. Use of DBS as a source of DNA for PCR could therefore be a convenient method for monitoring levels of malaria transmission in remote locations. As the results corresponded well to RDT results this may indicate that the most important and clinically relevant cases were found. The importance of the use of whole blood samples being significantly superior to DBS should be investigated further with studies of low-level parasitaemia using quantitative PCR or other quantitative techniques.

## Competing interests

The authors declare that they have no competing interests.

## Authors’ contributions

GEAS and BB were involved in all stages of this study. SM was involved in the design of the study and coordination of fieldwork. MF coordinated the fieldwork. NL was involved in the design of the study. All authors contributed to the data interpretation and writing of the manuscript. All authors have read and approved the final manuscript.
